# Cadmium inhibits hSMUG1-mediated uracil excision: quantitative analysis and mitigation by *Ganoderma lucidum* extracts

**DOI:** 10.1080/14756366.2026.2668792

**Published:** 2026-05-12

**Authors:** Hui-Lan Chang, Cheng-Hao Fang, Hsing-Lin Chang, Ya-Fen Lee, Wei-Lun Hsu, Jung-Hsuan Chang, Min-Hui Chien, Steven D. Goodman, Sui-Yuan Chang, Woei-horng Fang, Kang-Yi Su

**Affiliations:** ^a^ Department of Clinical Laboratory Sciences and Medical Biotechnology, College of Medicine, National Taiwan University, Taipei, Taiwan, ROC; ^b^ Genomic Research Center, Academic Sinica, Taipei, Taiwan, ROC; ^c^ Center for Microbial Pathogenesis, Nationwide Children’s Hospital and the Department of Pediatrics, Ohio State University, Columbus, OH, USA; ^d^ Department of Laboratory Medicine, National Taiwan University Hospital, Taipei, Taiwan, ROC; ^e^ Genome and Systems Biology Degree Program, College of Life Science, National Taiwan University, Taipei, Taiwan, ROC

**Keywords:** Cadmium, uracil excision, DNA repair, *Ganoderma*, hSMUG1

## Abstract

Cadmium is a pervasive environmental carcinogen known to disrupt multiple DNA repair pathways, yet its direct impact on uracil base excision repair enzyme activity remains unclear. Here, we quantitatively demonstrate that cadmium potently inhibits hSMUG1, a key enzyme initiating removal of mutagenic U:G mispairs arising from cytosine deamination. Using a nucleotide-resolution MALDI-TOF mass spectrometry assay, we show that cadmium suppresses hSMUG1 activity in a dose-dependent manner (IC_50_ = 4.6 μM), with near-complete inhibition at concentrations ≥25 μM. Notably, five Ganoderma lucidum extracts derived from different strains and extraction methods restored hSMUG1 activity (16.4–21.6%) under cadmium stress. ICP mass spectrometry revealed that these extracts reduced cadmium ion levels by 40–73%, suggesting metal chelation as a contributing mechanism. Together, our findings uncover a previously unrecognised mechanism by which cadmium compromises base excision repair and establishes a robust framework for evaluating environmental metal toxicity and its modulation at single-nucleotide resolution.

## Introduction

Environmental pollution by heavy metals such as cadmium, lead, arsenic, and mercury represent a pressing global health concern.^1^ These non-biodegradable elements accumulate in the environment and enter human body through the food chains, where they persist and exert long-term toxic effects.^2^ Chronic exposure to heavy metals has been strongly associated with a wide range of disease, including neurodegeneration, cardiovascular and renal dysfunction, and cancer.^3–6^ A key underlying mechanism is the induction of oxidative stress and genotoxicity, leading to DNA damage, replication stress, and disruption of DNA repair system.^7^ Notably, cadmium has been shown to impair DNA repair capacity and promote genomic instability, partly by inhibiting DNA repair enzymes and altering repair pathway choice.^7–10^

Despite increasing recognition of heavy metal–induced genotoxicity, the molecular mechanisms by which these toxicants interfere with specific DNA repair enzymes remain insufficiently characterised. In particular, quantitative assessment of repair activity at the enzymatic level under heavy metal exposure is technically challenging, and studies directly addressing how environmental toxicants affect base excision repair (BER) enzyme are still limited.

In parallel, natural products have attracted attention as potential protective agents against environmental toxicants. Among them, Ganoderma spp. (Lingzhi) has widely studied for its antioxidant, anti-inflammatory, immunomodulatory, hepatoprotective, and anticancer effects.^11–15^ It diverse bioactive components, including polysaccharides, triterpenoids, and phenolic compounds, have been implicated in mitigating oxidative stress and metal-associated toxicity.^16^ Previous studies have shown that Ganoderma extracts can reduce oxidative DNA damage and may interact with heavy metals through absorption or bioremediation mechanisms.^17–19^ For example, early evidence indicated that Ganoderma extracts could prevent DNA strand breaks caused by metal-catalysed Fenton reactions and ultraviolet irradiation, suggesting both direct and indirect DNA-protective effects.^20^ However, these observations are largely limited to indirect protective effects, and whether Ganoderma influences DNA repair processes, particularly at the level of specific repair enzymes under heavy metal-induced stress condition remains unclear.

Among DNA repair pathways, BER play a central role in removing small base lesions, including uracil generated through cytosine deamination or oxidative stress.^21^ Uracil misincorporation is mutagenic and, if not corrected, can lead to G:C to A:T transition mutations.^22^ This repair process is initiated by uracil-DNA glycosylases (UDGs), which excise uracil from DNA and thereby determine the efficiency of downstream repair. Among these enzymes, human single-strand selective monofunctional uracil-DNA glycosylase 1 (hSMUG1) is a key enzyme responsible for removing uracil and oxidised uracil derivatives under physiological conditions.^23^^,^^24^ Given its central role in maintaining genomic stability, impairment of hSMUG1 activity may compromise cellular defense against genotoxic stress. By preventing mutations caused by cytosine deamination and oxidative stress, hSMUG1 is a central component of genomic maintenance. Based on this rationale, we hypothesised that cadmium may disrupt uracil excision by directly inhibiting hSMUG1, and that Ganoderma extracts could preserve its repair activity under such conditions. To test this, we evaluated hSMUG1 activity using a non-labeled nucleotide MALDI-TOF mass spectrometry platform. In this study, we found that hSMUG1 exhibited substrate-dependent repair efficiency, while cadmium inhibited its activity in a dose-dependent manner (IC_50_ = 4.6 μM) and completely suppressed activity above 25 μM. Notably, under such inhibitory conditions, Ganoderma extracts restored hSMUG1 activity (16.4–21.6%) under inhibitory conditions, thereby improving BER. This approach establishes a standardised method to quantify hSMUG1 repair efficiency and support a potential role for Ganoderma in preserving BER capacity against heavy metal–induced genotoxicity.

## Results

### Quantification of hSMUG1-mediated uracil lesion excision by nucleotide mass spectrometry

**Figure 1.**
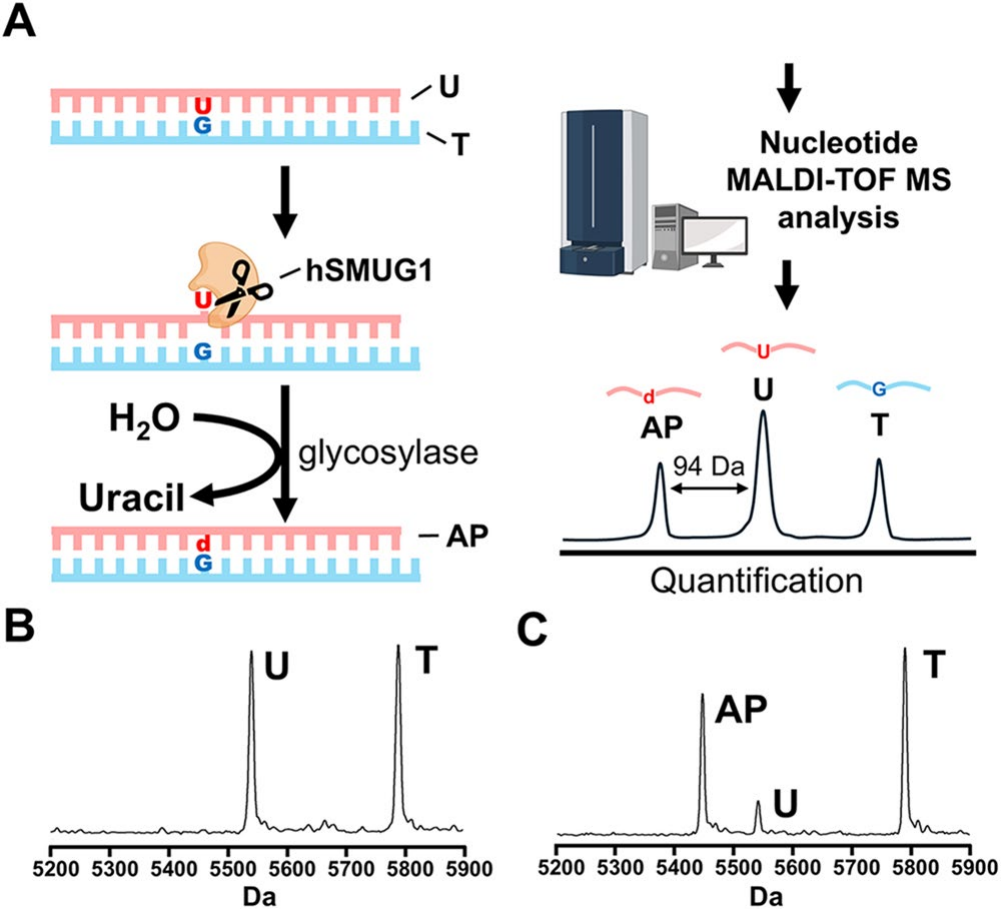
Quantitative analysis of hSMUG1-mediated uracil excision using nucleotide MALDI-TOF mass spectrometry. (A) Schematic representation of experimental design. A duplex DNA substrate was generated by annealing an 18-nucleotide lesion strand containing a signal uridine (U) with a complementary 19-nucleotide template strand (T), thereby introducing a U:G mispair at the centre of the duplex. Recombinant hSMUG1 catalysed the monofunctional glycosylase reaction, excising uracil to yield an abasic (AP site on the lesion strand called abasic primer (AP). The reaction products were subsequently profiled by nucleotide MALDI-TOF MS. Distinct m/z peaks corresponding to the U-, T-, and AP-containing strands enabled direct quantification of the excision reaction. Notably, uracil removal resulted in a 94 Da mass decrease, allowing precise discrimination between U and AP fragments. (B) In the absence of hSMUG1, the duplex exhibited two distinct peaks at 5789.8 Da (T strand) and 5541.6 Da (U strand) with high signal-to-noise ratios, confirming substrate integrity and mass resolution. (C) Upon hSMUG1 treatment, the uracil-containing (U) peak was predominantly converted into the AP peak at 5447.6 Da, indicating efficient excision of uracil from the U:G substate. Excision efficiency was determined from peak intensities using the formula: AP/(AP+U) × U x 100%.

To establish an *in vitro* system for evaluating and quantifying the efficiency of hSMUG1 in removing uracil (U) from DNA lesions, we employed a previously well-established nucleotide mass spectrometry platform.^25–27^ As an initial test and optimisation tool, we designed a synthetic substrate containing a U:G mispair (Figure 1(A)). This mispair was positioned in the middle of a duplex DNA formed by an 18-mer primer (U) annealed to a 19-mer template (T). During the reaction, hSMUG1 recognised the U and excised it via its glycosylase activity, thereby generating an abasic site (d) and leaving an abasic primer (AP). After terminating the reaction, nucleotide MALDI-TOF MS was used to analyse the m/z profiles of the nucleic acid fragments. In the absence of hSMUG1, clear signals were detected at 5541.6 Da and 5789.8 Da, corresponding to the U and T, respectively (Figure 1(B)). Upon the addition of hSMUG1, the U was excised, yielding an AP product signal at 5447.6 Da (Figure 1(C)). Based on the quantitative characteristics of mass spectrometry, the efficiency of hSMUG1-mediated U excision from the 18-mer primer was calculated using the formula AP/(AP+U) x U substrate amount, which served as the basis for subsequent analysis. Taken together, this method provides a robust and sensitive approach for assessing the uracil-removing activity of hSMUG1.

### *Optimisation of hSMUG1 enzyme concentration for uracil lesion excision* in vitro

**Figure 2.**
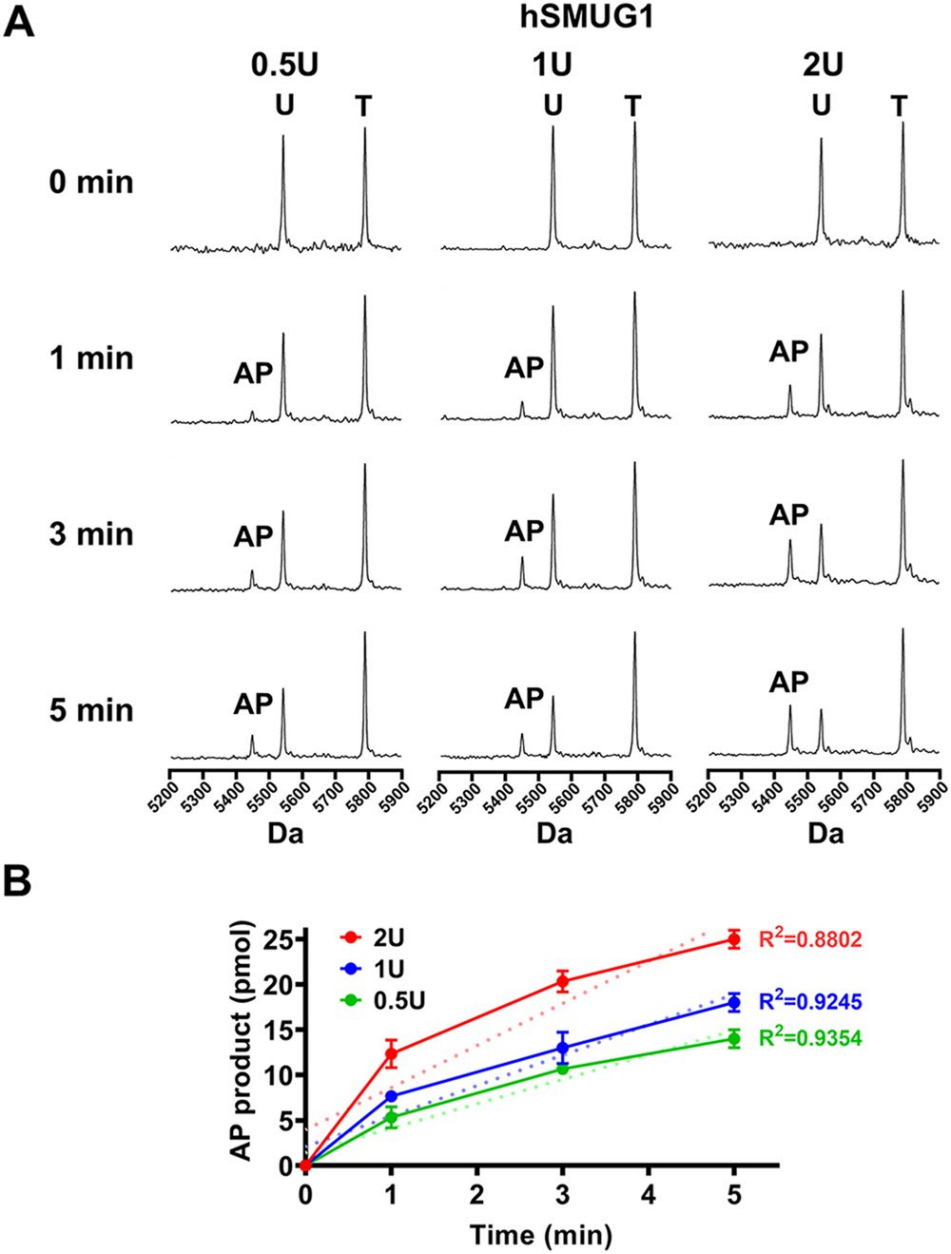
Evaluation of hSMUG1 concentration-dependent uracil excision activity. A 10 μL hSMUG1 glycosylase reaction mixture contained 50 pmol of uracil-containing duplex substrate (5 μM), 10 mM MgCl_2_, 100 μg/mL, 100 μg/mL bovine serum albumin (BSA), and 10 mM Bis-Tris Propane-HCl buffer (pH 7.0, 25 °C). Reactions were initiated by adding various concentrations of hSMUG1 (0.5–2 U, as indicated). (A) Representative MALDI-TOF mass spectra obtained from reactions at different enzyme concentrations and incubation times. Distinct peaks corresponding to the template strand (T), uracil-containing strand (U), and abasic primer (AP) enabled direct visualisation of uracil excision efficiency. (B) Quantitative analysis of AP primer formation. Each reaction was independently performed in triplicate for each concentration and time point. Data are presented as mean ± SD. The dashed line represents the best-fit linear regression curve describing the relationship between hSMUG1 concentration and uracil excision efficiency.

After establishing the *in vitro* quantitative assay for hSMUG1-mediated removal of uracil lesions in DNA, we next optimised parameters that could affect the measurement to provide suitable conditions for subsequent evaluation of cadmium effects. Regarding the optimal enzyme concentration and based on the commonly used 1 U *in vitro* enzymatic assays, we tested three concentrations of hSMUG1 (0.5 U, 1 U, and 2 U) to assess their uracil excision activity at different reaction times using 50 pmol of the U-primer substrate as input (Figure 2(A)). The results showed that AP products were detectable as early as 1 min at all enzyme concentrations, and their levels increased in a time-dependent manner up to 3 and 5 min. Triplicate experiments followed by quantitative and linear regression analyses further revealed a clear dosage-dependent effect of hSMUG1 on AP product formation at different time points, with *R*^2^ values of 0.8802, 0.9245, and 0.9354 for 2 U, 1 U, and 0.5 U, respectively (Figure 2(B)). Considering both excision efficiency and the linearity between AP product formation and reaction time, 1 U hSMUG1 was selected for the optimal concentration for subsequent assays.

### Evaluation of hSMUG1 excision efficiency on uracil-containing mispairs

**Figure 3.**
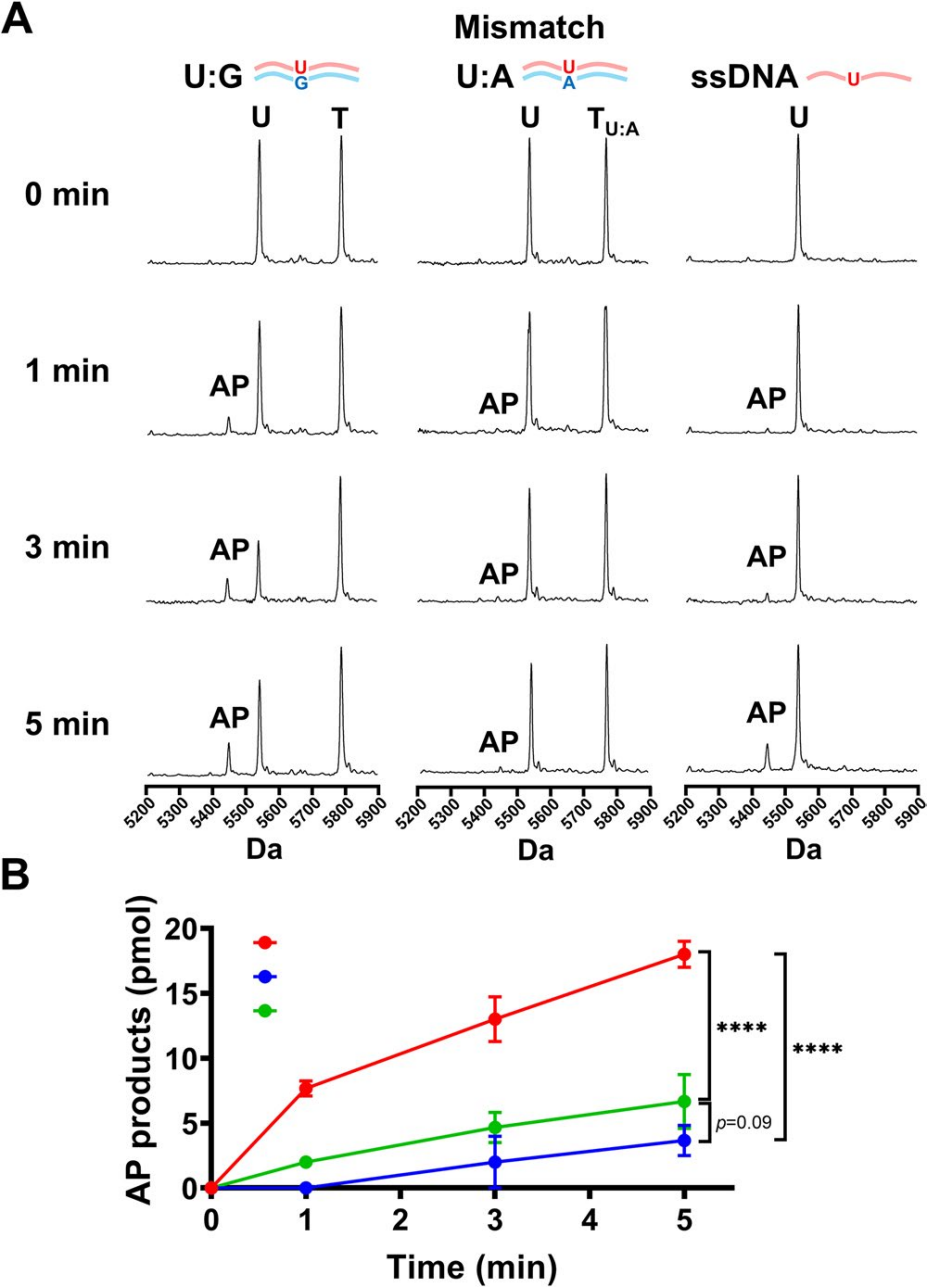
Evaluation of hSMUG1-mediated uracil excision efficiency in damaged partners duplex or single-stranded DNA. A 10 μL hSMUG1 glycosylase reaction mixture contained 1 U hSMUG1, 10 mM MgCl_2_, 100 μg/mL, 100 μg/mL bovine serum albumin (BSA), and 10 mM Bis-Tris Propane-HCl buffer (pH 7.0, 25 °C). Reactions were initiated by adding various 50 pmol (5 μM) of uracil-containing substrates, including U:G or U:A mispair in the duplex DNA, or uracil in single-stranded DNA (ss U). (A) Representative MALDI-TOF mass spectra of reactions with different substrates and incubation times. Distinct peaks corresponding to the template strand (T or T_U:A_), uracil-containing strand (U), and abasic product (AP) allowed direct visualisation of uracil excision efficiency. (B) Quantitative analysis of AP product formation. Each reaction was independently performed in triplicate for each substrate and time point. Data are presented as mean ± SD. Statistical analyses were performed by two-way ANOVA with Geisser-Greenhouse correction. ^****^p < 0.0001.

Based on the previous results, we next examined whether the base-pairing partner affects hSMUG1 recognition and uracil excision. Under physiological condition, DNA is subjected to stresses such as oxidative radicals, radiation, or chemical agents, which can induce cytosine deamination, generate uracil and result in U:G mispairs. If hSMUG1 fails to recognise and remove these lesions in time, U:A mismatches may arise during DNA replication. To investigate substrate preference, we designed three types of substrates—U:G, U:A, and single-stranded DNA containing uracil—to mimic post-damage repair scenarios and evaluate which substrate is most efficiently recognised and excised by hSMUG1 (Figure 3). The results showed that AP products generated from U:G lesions increased in a time-dependent manner at 1, 3, and 5 min. (Figure 3(A)). Triplicate reactions followed by quantitative analysis of AP production revealed that U:G mispair exhibited a clear time-dependent increase, and at 5 min. The AP product levels generated from the U:A and single-stranded U substrates were significantly lower than those from the U:G lesion group; however, there was no significant difference between the U:A and single-stranded U substrate groups by two-way ANOVA analysis with Geisser-Greenhouse correction (Figure 3(B)). In summary, this result indicates that U:G paired substrates should be prioritised in subsequent hSMUG1 activity assays.

### Impact of uracil lesion position on hSMUG1 excise efficiency

**Figure 4.**
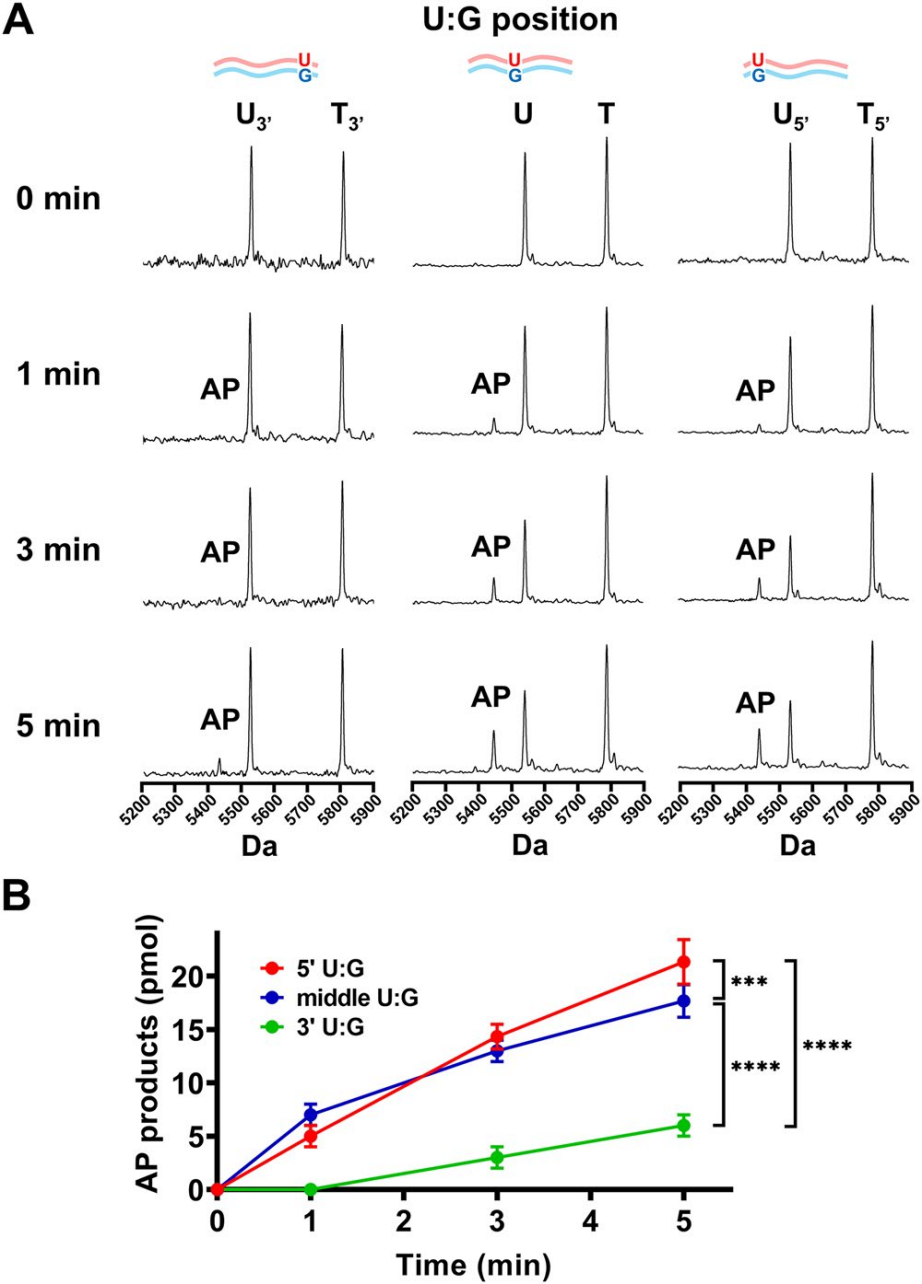
hSMUG1-mediated excision efficiency of U:G mispair at different positions within duplex DNA. A10 μL hSMUG1 glycosylase reaction mixture contained 1 U hSMUG1, 10 mM MgCl_2_, 100 μg/mL, 100 μg/mL bovine serum albumin (BSA), and 10 mM Bis-Tris Propane-HCl buffer (pH 7.0, 25 °C). Reactions were initiated by adding various 50 pmol (5 μM) of substrates containing U:G mispair positioned at the 3′ end, middle, or 5′ end of duplex DNA. (A) Representative MALDI-TOF mass spectra of reactions with different substrates and incubation times. Distinct peaks corresponding to the template strand (T or T_3′_ or T_5′_), uracil-containing strand (U or U_3′_ or U_5′_), and abasic primer (AP) allowed direct visualisation of uracil excision efficiency. (B) Quantitative analysis of AP product formation. Each reaction was independently performed in triplicate for each substrate and time point. Data are presented as mean ± SD. Statistical analyses were performed by two-way ANOVA with Geisser-Greenhouse correction. ***p < 0.001; ^****^p < 0.0001.

In the final stage of the pilot test, we sought to determine whether the position of the U:G mispair within the synthetic DNA substrate affects the uracil excision efficiency of hSMUG1. To this end, three substrates were synthesised, each containing a U:G mispair located at distinct positions: near the 3′ end (3 base pairs from the terminus), in the middle, or near the 5′ end (3 base pairs from the terminus) of the duplex (Figure 4). The results showed that when the U:G mispair was positioned in the middle or near the 5′ end, AP products accumulated in time-dependent manner as the reaction progressed. In contrast, when the mispair was located near the 3′ end, detectable AP products appeared only after 5 min of incubation (Figure 4(A)). Quantitative analysis of triplicate reactions further revealed that uracil excision by hSMUG1 was significantly more efficient when the mispair was positioned in the 5′ end than when it was near the 3′ end or the middle positions by two-way ANOVA analysis with Geisser-Greenhouse correction (Figure 4(B)). Taken together, these results indicate that the positional context of the U:G mispair influences hSMUG1 activity. Therefore, for subsequent experiments assessing cadmium-induced inhibition, the substrate containing a centrally located U:G mispair under the optimised pilot conditions was selected for further analysis.

### Concentration-dependent analysis of cadmium impact on hSMUG1 uracil lesion excision

**Figure 5.**
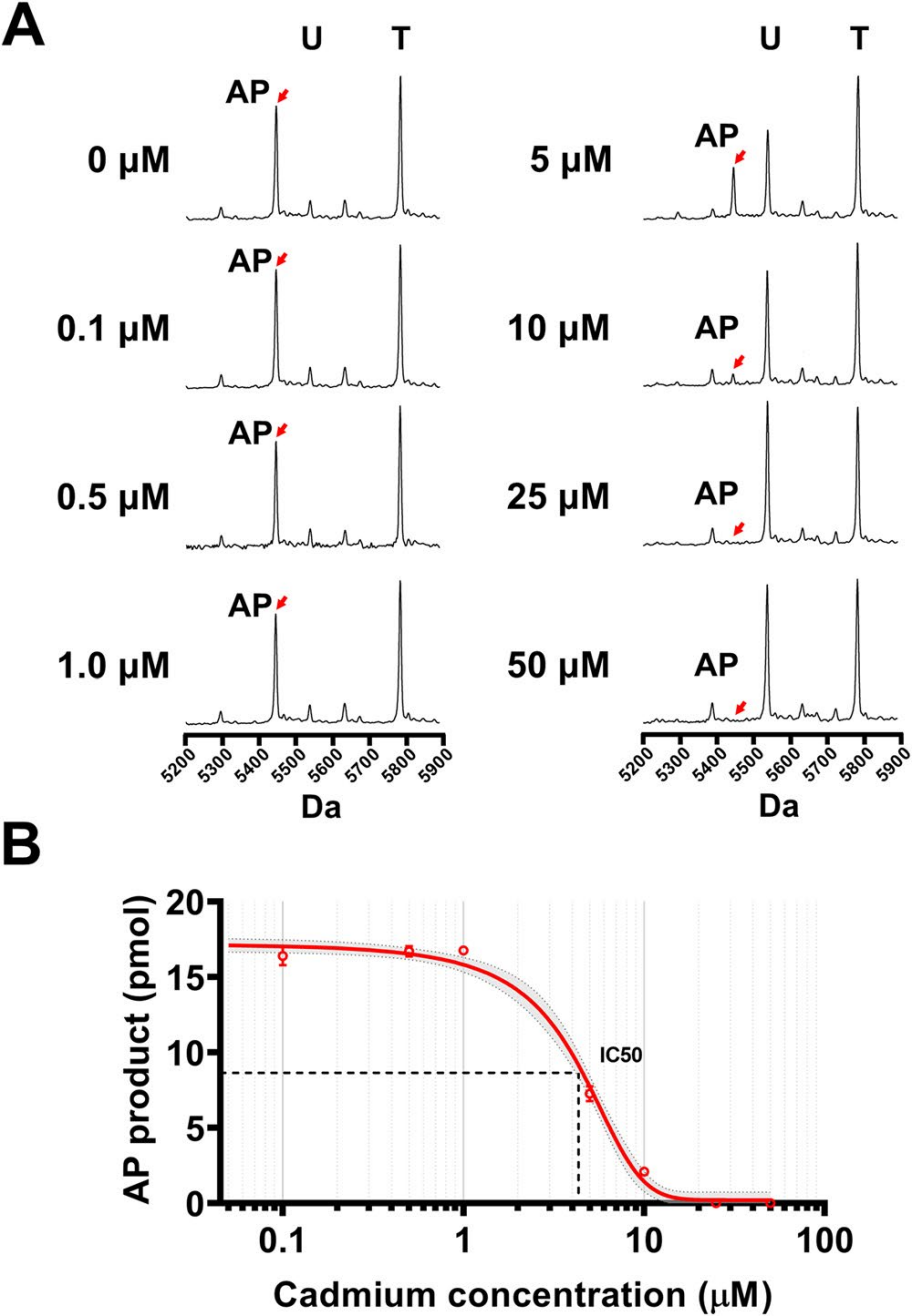
Effect of cadmium on hSMUG1-mediated uracil excision from DNA. A10 μL hSMUG1 glycosylase reaction mixture contained 1 U hSMUG1, 10 mM MgCl_2_, 100 μg/mL, 100 μg/mL bovine serum albumin (BSA), and 10 mM Bis-Tris Propane-HCl buffer (pH 7.0, 25 °C). Reactions were initiated by adding 20 pmol substrate DNA in the presence of cadmium at various concentrations (0–50 μM). (A) Representative MALDI-TOF mass spectra of reactions with different cadmium concentrations and incubation times. Distinct peaks corresponding to the template strand (T), uracil-containing strand (U), and abasic primer (AP) allowed direct visualisation of uracil excision efficiency. (B) Inhibition curve of hSMUG1 activity by cadmium. Triplicate reactions performed under each cadmium concentration were used to generate an inhibition curve. The IC_50_ of cadmium for hSMUG1 inhibition was 4.6 μM. The red arrow indicates the peak corresponding to the AP product. Data are presented as mean ± SD.

We hypothesised that the presence of cadmium may inhibit hSMUG1-mediated excision of uracil generated by cytosine deamination following DNA damage, thereby impairing subsequent repair processes. To test this, various concentrations of cadmium were added to the *in vitro* hSMUG1 reaction mixture containing the U:G mispair DNA to assess whether AP product formation decreased upon uracil excision (Figure 5). In the absence of cadmium, 84.8% of uracil in the initial 20 pmol U-primer substrate was removed, yielding approximately 16.7 pmol of AP products. As cadmium concentration increased, AP product levels progressively declined in a dose-dependent manner (Figure 5(A)). Triplicate reactions performed under each cadmium concentration were used to generate an inhibition curve (Figure 5(B)). The results revealed that the IC_50_ of cadmium for hSMUG1-catalysed uracil excision was approximately 4.6 μM. Notably, at cadmium concentrations above 25 μM, hSMUG1 activity was completely abolished, and no detectable AP products were observed. Based on these findings, 25 μM cadmium was selected for subsequent experiments to evaluate whether Ganoderma extracts could rescue hSMUG1 activity.

### Protection of hSMUG1 glycosylase activity by Ganoderma extracts through reducing cadmium residue

**Figure 6.**
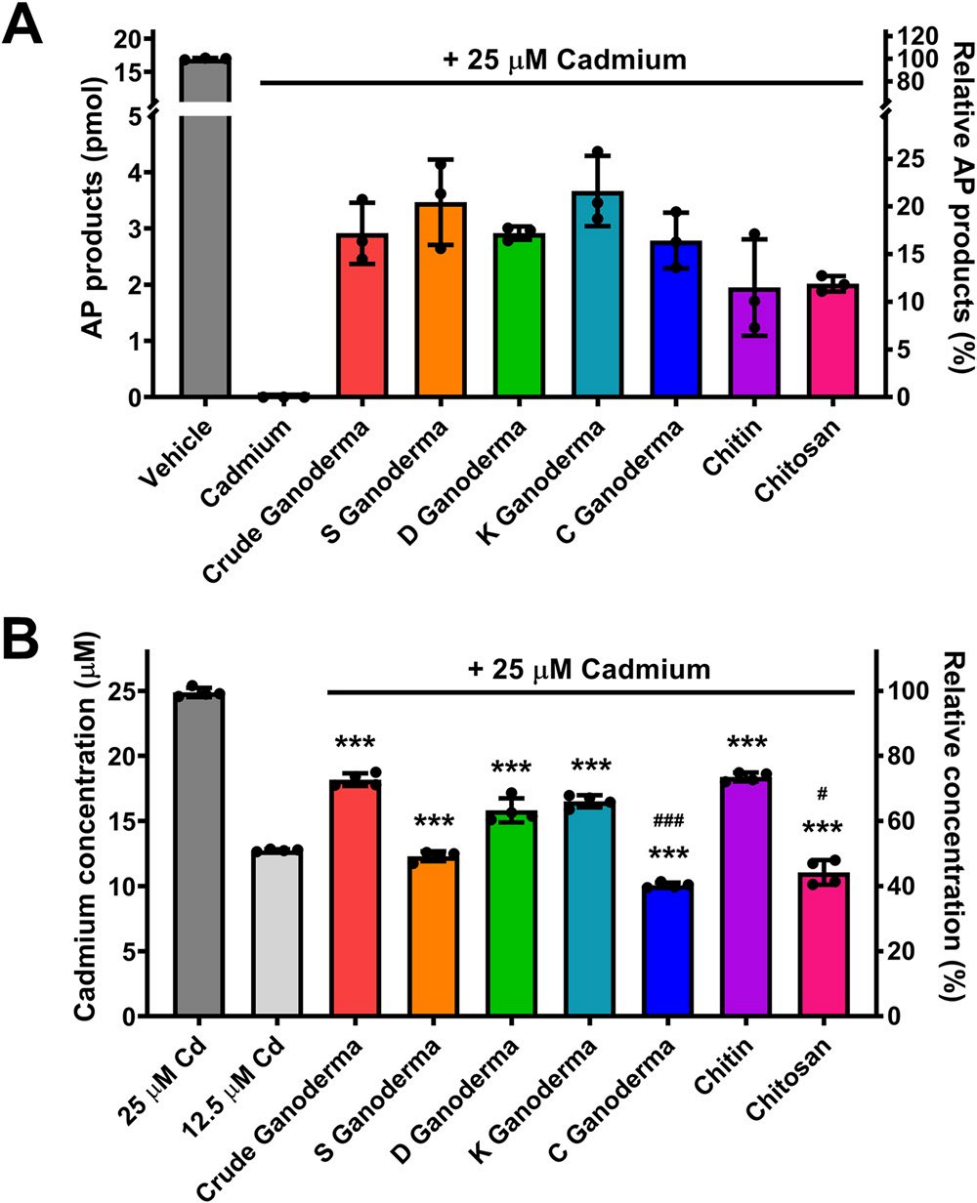
Evaluation of the restorative and neutralising effects of Ganoderma extracts on cadmium-inhibited hSMUG1 activity. In uracil excision reactions, 25 μM cadmium was used as a baseline to establish complete inhibition of hSMUG1 activity. To assess the recovery effects, different Ganoderma extracts (25 mg/L) were tested including Crude Ganoderma (powdered YK-01 fruiting body), S Ganoderma (water and ethanol extracts of YK-01 fruiting body), K Ganoderma (water extract of YK-02 fruiting body), C Ganoderma (water and ethanol extracts of YK-07 fruiting body), and D Ganoderma (a mixture of 90% water and ethanol extracts from fresh YK-01 fruiting body and 10% dried Ganoderma formosanum). (A) Quantitative analysis of the recovery effects of Ganoderma extracts on hSMUG1 activity. (B) Quantification of cadmium neutralisation by Ganoderma extracts. Each extract (25 mg/L) was incubated with 25 μM cadmium, followed by filtration; the remaining cadmium concentration in the filtrate was determined by inductively coupled plasma-mass spectrometry (ICP-MS). Chitosan and chitin were included as representative controls for water-soluble and insoluble Ganoderma constituents, respectively. Each reaction was independently performed in triplicate for each extract. Data are presented as mean ± *SD*. ***P < 0.001 compared with 25 μM cadmium control; ^#^*P* < 0.05 compared with 12.5 μM cadmium; ^###^*P* < 0.001 compared with 12.5 μM cadmium.

We next examined whether Ganoderma extracts could restore hSMUG1 activity inhibited by cadmium. In this assay, 5 μL of each Ganoderma extract solution was added to the *in vitro* reaction mixture, resulting in a final concentration equivalent to the water-soluble fraction of 25 mg/L Ganoderma products, followed by quantification of AP product formation (Figure 6(A)). Five Ganoderma extracts, including crude Ganoderma, S Ganoderma, D Ganoderma, K Ganoderma, and C Ganoderma, were tested for their ability to rescue hSMUG1 activity. In addition, the water-soluble and insoluble components of Ganoderma, chitosan (0.5 g/L) and chitin (0.5 g/L), were included as control groups. In the absence of cadmium, hSMUG1 concerted 20 pmol of U-primer substrate into 16.9 pmol of AP products. The presence of 25 μM cadmium completely inhibited hSMUG1 activity, eliminating AP formation. Both chitosan and chitin partially restored hSMUG1 activity, yielding 11.8 ± 0.8% and 11.5 ± 5.1% of AP products, respectively. Under the same inhibitory conditions (25 μM cadmium), all five Ganoderma extracts effectively rescued hSMUG1 activity, with AP production restored to varying degrees. Among them, K Ganoderma showed the strongest effect (21.6 ± 3.7%), followed by S Ganoderma (20.4 ± 4.5%).

To further determine whether Ganoderma extracts neutralise or block the toxic effect of cadmium, each extract was incubated with cadmium prior to filtration, and the remaining cadmium concentration was quantified by ICP-mass spectrometry (Figure 6(B)). Using 25 μM and 12.5 μM cadmium as reference controls, chitosan and chitin significantly reduced cadmium levels (residual cadmium = 11.1 ± 0.8 μM and 18.4 ± 0.3 μM, respectively). Similarly, all Ganoderma extracts markedly decreased cadmium concentrations, with C Ganoderma showing the strongest neutralising effect (residual cadmium = 10.1 ± 0.2 μM), followed by S Ganoderma (12.3 ± 0.3 μM). Collectively, these results demonstrate that Ganoderma extracts can neutralise approximately 50% of cadmium and thereby restore about 20% of the hSMUG1 activity otherwise inhibited by cadmium. These findings suggest that Ganoderma extract effectively protects hSMUG1 from cadmium-induced inhibition, enabling the enzyme to excise uracil from damaged DNA and prevent mutagenic mispairing during subsequent replication.

## Materials and methods

### Oligonucleotide substrates and reagents

Synthetic oligonucleotides purified by HPLC were purchased from Integrated DNA Technologies (Singapore). The sequences of the nucleic acid used in this study are in Table 1 (Table 1). The powder form of DNA was dissolved in 10 mM Tris-HCl and 1 mM EDTA (TE) to make 100 μM stock and stored at −20 °C. Cadmium chloride hemipentahydrate (CdCl_2_‧5/2 H_2_O, Mw 228.36) was from Sigma-Aldrich and dissolved in sterilised water to make a 20 mM stock and stored at room temperature.

**Table 1.** Oligonucleotides used for substrate preparation.

ID	Sequences^a^	Mass (Da)
Template strand (T) (for U:G)	3′-CTGGTCAGGCCTGCAACCG	5789.8
Template strand (T_U:A/_T_5′_) (for U:A)	3′-CTGGTCAGACCTGCAACCG	5773.8
Template strand (T_3′_)	3′-CTGGTCAGGCCTGCAGCCG	5805.8
Uracil contained primer (U)	5′-GACCAGTC**U**GGACGTTGG	5541.6
Uracil contained primer (U_5′_)	5′-GA**U**CAGTCCGGACGTTGG	5541.6
Uracil contained primer (U_3′_)	5′-GACCAGTCCGGACGT**U**GG	5526.6

^a^ Uracil bases are in bold.

Human single-strand selective Uracil-DNA Glycosylase (hSMUG1) and companion buffers were purchased from New England Biolabs, MA. The hSMUG1 (5000 U/mL, 167 nM) used in this study followed manufacturer’s unit definition and the following enzyme dilution buffer: 50% glycerol, 250 mM NaCl, 0.1 mM EDTA, 1 mM dithiothreitol, 200 μg/ml recombinant albumin,0.15% Triton^®^ X-100, 10 mM Tris-HCl (pH 7.4 @25 °C).

### Ganoderma extracts preparation

All Ganoderma extracts used in this study were provided by Dr. Deng-Hai Chen (Double Crane Enterprise, Taiwan). A total of five extracts was prepared from Ganoderma lucidum fruiting bodies (strains YK-01, YK-02, and YK-07) using either hot water or ethanol extraction. The extracts included: Crude Ganoderma (powered YK-01 fruiting body), S Ganoderma (water and ethanol extracts of YK-01 fruiting body), K Ganoderma (water extract of YK-02 fruiting body), C Ganoderma (water and ethanol extracts of YK-07 fruiting body), and D Ganoderma (comprising 90% water and ethanol extracts of fresh YK-01 fruiting body and 10% dried Ganoderma formosanum). The detailed composition of each extract, including extraction yield and major constituent profiles, is summarised in Table 2 (Table 2).

**Table 2.** Definition and preparation of Ganoderma extracts used in this study.

Extract Name	Source Strain	Extract Method	Description	Extraction Yield (% of dry weight)	Major Components (approximate content)
Crude Ganoderma	YK-01	None (raw material)	Power of the fruiting body	6.2	1% ganoderic acids; 5% polysaccharides
S Ganoderma	YK-01	Water and ethanol extraction	Combined water and ethanol extracts of the fruiting body	12.0	3% ganoderic acids; 8% polysaccharides
K Ganoderma	YK-02	Water extraction	Water extract of the fruit body	5.0	4% lucidenic acids
C Ganoderma	YK-07	Water and ethanol extraction	Combined water and ethanol extracts of the fruiting body	5.0	1% ganoderic acids; 4% polysaccharides
D Ganoderma	YK-01/Ganoderma formosanum	Water and ethanol extraction	Extracts derived from a mixture of YK-01 (fresh, 90%) and Ganoderma formosanum (dried, 10%) fruiting bodies	6.0	5% ganoderic acids; 6% polysaccharides

For hot water extraction, dried fruiting bodies of Ganoderma lucidum were cut into small pieces and ground into coarse powder. The powder was extracted with distilled water by boiling at 90–100 °C for 2–4 h. The aqueous extract was filtered to remove insoluble residues, and the filtrate was concentrated under reduced pressure. The resulting precipitate was collected by centrifugation, washed with ethanol, and lyophilised for subsequent analyses. For ethanol extraction, powdered fruiting bodies of Ganoderma lucidum were extracted with 70–95% (v/v) ethanol at room temperature or under reflux for 24–72 h. Insoluble materials were removed by filtration, and the filtrate was concentrated under reduced pressure using a rotary evaporator. The concentrated extract was further dried to obtain crude ethanol-soluble fractions.

### hSMUG1 DNA glycosylase reaction

For hSMUG1 DNA glycosylase reaction, 50 pmol duplex DNA was used for characterizing hSMUG1 initial rate and 20 pmol duplex DNA was used for inhibition assays. Equal molar amounts of template and lesion containing DNA were mixed and incubated at 65 °C for 30 min, then at 37 °C for 30 min, and finally on ice to ensure proper annealing. A 10 μL glycosylase reaction mixture contained 20–50 pmol of uracil substrate, 10 mM MgCl_2_, 100 μg/mL bovine serum albumin, and 10 mM Bis Tris Propane-HCl (pH 7.0 at 25 °C). Reactions were started by the addition of different concentrations of hSMUG1 from 0.5 U to 2 U as indicated for each experiment. The uracil hydrolysis reaction was performed at 37 °C and quenched by adding 1 μL 0.25 M HCl on ice for 6 min, dropping the pH to < 2 and stopping the reaction. The pH of the mixture was then neutralised with 1 μL 0.23 M tris base. The mixtures were diluted with 13 μL TE then stored at −20 °C prior to MALDI-TOF MS analysis. AP-products were subjected to MS analysis within 30 h to minimise spontaneous hydrolysis of AP-sites to an undetectable level.

### Nucleotide MALDI-TOF MS analysis

The analysis and quantification of uracil excision by hSMUG1 using nucleotide MALDI-TOF MS were performed based on our previous studies with modifications to meet the requirements of this study.^25–28^ All procedures were conducted in a certified clinical laboratory, Pharmacogenomic Lab, College of Medicine, National Taiwan University. Briefly, the reaction products were sequentially desalted using Sephadex G-10 (Pharmacia, Sweden) and resin prior to MALDI-TOF MS analysis. Sephadex G-10 power was pre-treated as follows: 100 g of powder was hydrated with 250 ml of Tris-EDTA buffer, followed by removal of the supernatant. The resin was suspended with an equal volume of Tris-EDTA buffer and washed for three times to remove impurities. The hydrated power was autoclaved and stored at 4 °C until use. For desalting, the reaction mixture was adjusted to a final volume of 100 μL and applied to the column, which was placed in an Eppendorf. Desalted reaction products were collected by centrifugation at 600 × g for 4 min and stored at −20 °C before subsequent resin desalting and MALDI-TOF MS analysis.

For MALDI-TOF MS analysis, the desalted product was transferred to a 384-well plate and spotted onto a SpectroCHIP Array using a nanodispenser (Agena Bioscience, CA, USA). The analyses were performed using MassARRAY System (Agena Bioscience, CA, USA) according to the manufacturer’s instructions with minor modifications.^28^ Briefly, for assay setup, files containing the m/z information of anticipated signals, such as uracil-containing nucleotide substrates, templates, and AP-site-containing nucleotide products, were prepared and imported into the Assay Designer module of the Type 4 software. The application assigned each assay to its corresponding chip position and generated a working list for all samples. Raw data were generated by Type4 software. The mass spectra graph could be customised by the analysis program, with the X-axis representing the range of m/z ratio and the Y-axis showing the upper and lower limits of signal peak intensity. Mass graphs exported with setting the specific range of m/z including substrate, products, and template intensity peaks. The peak intensity measured for enzyme activity determination. The enzyme converted-AP product calculation formula as below:

$$\text{Gly}\text{cos}\;yl\text{ase}\;\text{activity}=\frac{\text{AP}\;\text{product}\;\text{signal}\;}{U\;\text{substrate}\;\text{signal}\;+\text{AP}\;\text{product}\;\text{signal}\;}\times U\;\text{substrate}$$

The relative peak intensities were measured to determine enzyme activity.

### Cadmium inhibition assay and Ganoderma extracts antitoxic assay

For *Ganoderma* extracts solution preparation, 0.5 g of each *Ganoderma* product was grinded with pestle and dissolved in 100 ml sterilised H_2_O. The suspension was then transferred into a roller bottle and rotated for 1 h. The suspension was then centrifuged 1000xg for 10 min, and the supernatant was collected and filtered through a 0.22 μm filter. The *Ganoderma* extract solution was store and stable at 4 °C for 1 month.

For the cadmium inhibition assay, hSMUG1 was incubated in reaction buffer containing different concentrations of cadmium on ice for 10 min. Reactions were started by adding 20 pmol duplex DNA in a 10 μl reaction. Determination of hSMUG1 activity was performed as described above. The inhibition curve was determined by data fitting with the online program “Quest Graph^™^ IC_50_ Calculator.” AAT Bioquest, Inc. https://www.aatbio.com/tools/ic50-calculator.

For *Ganoderma* extracts antitoxin protection assays, 5 μl of each Ganoderma extracts solution (the final concentration was relative to water-soluble fraction of 25 mg/L Ganoderma products) was mixed with 25 μM of cadmium in reaction buffer on ice for 10 min. Reactions were started by adding 20 pmol substrate DNA in a 10 μl reaction. Determination of hSMUG1 DNA glycosylase activity was performed as described above.

### ICP mass spectrometry assay for residual cadmium quantification

For residual cadmium quantification after Ganoderma extract incubation, 0.5 g of each Ganoderma product, chitosan, and chitins was grinded with pestle and dissolved in 100 ml 50 μM cadmium solution prepared in ultrapure water. The suspensions were incubated in roller bottles and rotated for 1 h at room temperature to facilitate cadmium binding. Following incubation, samples were centrifuged at 1000 × g for 10 min, and the supernatant were collected and filtered through a 0.22 μm membrane filter to remove particulate material. Prior to inductively coupled plasma-mass spectrometry (ICP-MS) analysis, filters were diluted as necessary and acidified with trace metal-grade nitric acid to a final concentration of approximately 1% (v/v) to stabilise dissolved cadmium and minimise adsorption losses.

Cadmium concentrations were determined at the Department of Laboratory Medicine, National Taiwan University Hospital, an ISO 15189-certified clinical laboratory, using an ICP-MS (NexION 1000 G, PerkinElmer) operated under validated condition for trace metal analysis. Quantification was performed using an external calibration curve constructed from commercially available cadmium standard solutions prepared in a matrix-matched nitric acid solution (1% v/v). The calibration curve typically consisted of multiple concentration points spanning the expected analytical range, and linearity was confirmed with a correlation coefficient (R^2^) exceeding 0.995.

To ensure accuracy and compensate for potential matrix effects and instrumental drift, an internal standard ((e.g., indium,^115^In) was continuously introduced into all samples, calibration standards, and blanks at a fixed concentration. The internal standard signal was used to normalise analyte responses and correct signal fluctuations during analysis. Calibration verification standards were analysed periodically throughout the analytical run to confirm calibration stability and accuracy. Analytical performance was further evaluated through routine quality control procedures, including the use of procedural blanks, duplicate measurements, and repeat analyses. Where applicable, recovery and consistency of measured concentrations across replicates were assessed to ensure acceptable precision and reproducibility. Potential spectral interferences were minimised by appropriate instrument tuning and the use of collision cell technology in helium mode, following the manufacturer’s recommendations. Al samples were analysed in triplicate at least, and mean values were used for subsequent analysis. The overall method performance, including linearity, signal stability, and reproducibility, met the acceptance criteria for trace metal quantification in clinical ICP-MS practice.

### Statistical analysis

All data are presented as mean ± standard deviation (SD). Statistical analyses were performed using GraphPad Prism (version 8.0). For comparisons between two groups at a single endpoint, an unpaired Student’s *t*-test was applied. For experiments involving two independent variables (time and experimental group), data were analysed using two-way analysis of variance (two-way ANOVA), with *time* and *group* as factors. The interaction term (time × group) was included to evaluate whether temporal trends differed among groups. When appropriate, *post hoc* multiple comparisons were conducted with Geisser-Greenhouse correction for multiple testing. Exact *p* values are reported where applicable, and a *p* values < 0.05 was considered statistically significant.

## Discussion

Ganoderma lucidum has been widely studied for its diverse biological activities in East Asia for several thousand years. It has been extensively applied in health maintenance and as an adjunctive therapy in clinical practice.^29^ Modern pharmacological studies have confirmed that Ganoderma possesses multiple biological activities, including antioxidant, anti-inflammatory, immunomodulatory, antitumor, and hepatoprotective effects.^30–34^ Its major bioactive constituents, such as polysaccharides, ganoderic acid, various alkaloids, and protein, are believed to exert their effects through distinct cellular signalling pathways. In this study, rather than broadly emphasising its pharmacological properties, we specifically investigated whether Ganoderma extracts can impact the activity of a defined DNA repair enzyme under conditions of heavy metal stress. Previous reports have suggested that Ganoderma extracts may protect DNA from damage induced by γ-irradiation or reactive oxygen species, yet such findings were primarily based on macroscopic assays such as the comet assay, which cannot precisely resolve or quantify the excision and repair of damaged bases.^35–37^ Here, we applied nucleotide mass spectrometry for the first time to directly quantify hSMUG1-mediated uracil excision, enabling precise measurement of enzymatic activity at single-nucleotide resolution. This methodological approach provides a robust framework for evaluating how environmental toxicants, such as cadmium, influence base excision repair processes at the enzymatic level.

A central finding of this study is the pronounced biochemical sensitivity of hSMUG1 to cadmium. Our results demonstrate that cadmium inhibits hSMUG1-mediated uracil excision in a dose-dependent manner, with an IC_50_ of approximately 4.6 μM and near-complete inhibition at concentrations ≥ 25 μM. These findings suggest that hSMUG1 is highly susceptible to disruption by cadmium at concentrations relevant to environmental and biological exposure.^38^^,^^39^ Given that hSMUG1 catalyses the initiating step of the BER pathway, its inhibition would be expected to impair downstream repair processes and increase the likelihood of mutation fixation during DNA replication.^40^^,^^41^ Consistent with this role, our substrate analysis further demonstrates that hSMUG1 preferentially excises uracil from U:G mispair compared with U:A mispair or single-stranded substrates (Figure 3), highlighting its importance in preventing cytosine deamination-induced mutagenesis. Together, these findings support a mechanism by which cadmium may contribute to genomic instability, at least in part, through direct inhibition of hSMUG1 activity.

Within this context, the observed effects of five different Ganoderma extracts (crude, S, K, C, and D types) can be interpreted in mechanistic terms. Although the precise identities and concentrations of the active compounds remain to be determined, our data suggest that the partial restoration of hSMUG1 activity is primarily associated with a reduction in bioavailable cadmium. This interpretation is supported by ICP-MS analysis showing a substantial decrease in cadmium levels following incubation with Ganoderma extracts, as well as by the partial protective effects observed with chitosan and chitin, which are representative structural compounds of Ganoderma. This raises the possibility that the rescue effect on hSMUG1 activity could be due to known major constituents (such as polysaccharides, triterpenoids, sterols, and proteins) or to less studied minor components.^16^^,^^42^ Their ability to mitigate cadmium inhibition suggests that polysaccharide or amine/amine-bearing polymers may have a role, for example by binding or chelating cadmium, thereby reducing its bioavailability to hSMUG1^43^. Nonetheless, the fact that Ganoderma extracts outperformed chitosan or chitin alone in restoring hSMUG1 activity indicates that there are additional factors (other active compounds) contributing to this effect. Further studies will be required to identify the key bioactive components and to determine whether mechanisms beyond metal sequestration may also contribute to the observed effects.

This study further shows that Ganoderma extracts can restore hSMUG1 activity under cadmium-containing environments, allowing the enzyme to continue removing uracil from damaged DNA through the BER pathway. Given that hSMUG1 functions as an initiating role in maintaining genome integrity, its inhibition may lead to inefficient uracil excision and increased accumulation of G:C to A:T transition mutations. Such alterations have been associated with genomic instability and have been reported in various tumors.^44^^,^^45^ In addition, spontaneous cytosine deamination is estimated to occur approximately 100–500 times per day in living cell,^41^ suggesting that sustained impairment of uracil-DNA glycosylase activity could contribute to mutation accumulation over time. These findings are based on *in vitro* observations and support a potential mechanistic link between cadmium-induced inhibition of hSMUG1 and impaired uracil excision. On the other hand, under conditions of chronic exposure to environmental toxicants such as heavy metals, hSMUG1 activity may be compromised, potentially leading to reduced DNA repair and an increased mutational burden.^46^^,^^47^ In this context, our finding that cadmium at environmentally and physiologically relevant concentrations inhibits hSMUG1 activity^38^^,^^39^ provides a possible mechanistic explanation for its genotoxic effects. Conversely, the observation that Ganoderma extracts attenuate this inhibition suggests a potential role in preserving DNA repair activity under stress conditions. However, these findings are based on *in vitro* assays, and further studies are required to determine whether similar effects occur in physiological settings.

Several limitations of this study should be acknowledged. First, regarding the repair process of DNA substrates induced by DNA damage, our investigation focused exclusively on uracil excision; subsequent steps, including incision of the DNA backbone 5′ to the AP site, removal of the 5′-deoxyribose phosphate residues, extension of the 3′-OH, and nick sealing, were not addressed. Second, given the inherent challenges in functionally assessing base excision repair *in vivo*, this study was limited to *in vitro* enzymatic assays using purified components; future studies will need to overcome technical hurdles to validate these findings in living systems. Third, although the identity of the mismatched base opposite uracil and the location of the lesion within the DNA duplex influenced hSMUG1-mediated uracil excision efficiency, the potential impact of neighbouring nucleotide sequences could not be excluded and remains unresolved based on the current data. Finally, although Ganoderma extracts partially restored hSMUG1 activity under cadmium exposure, the present data do not distinguish whether this effect results from direct modulation of hSMUG1 or indirectly from reduced cadmium bioavailability. Our ICP-MS results support a mechanism involving decreased free cadmium levels, thereby alleviating inhibition of hSMUG1. However, a direct interaction between Ganoderma-derived compounds and hSMUG1 cannot be established in the current study. In addition, the functional consequences of altered DNA repair efficiency are highly context-dependent. Although variations in DNA repair capacity have been associated with processes such as cellular stress responses, tumorigenesis, and ageing, these relationships cannot be directly inferred from the present *in vitro* findings. Further investigation will be required to clarify the underlying mechanisms and to determine the biological relevance of these observations in more complex biological systems.

## Conclusion

**Figure 7.**
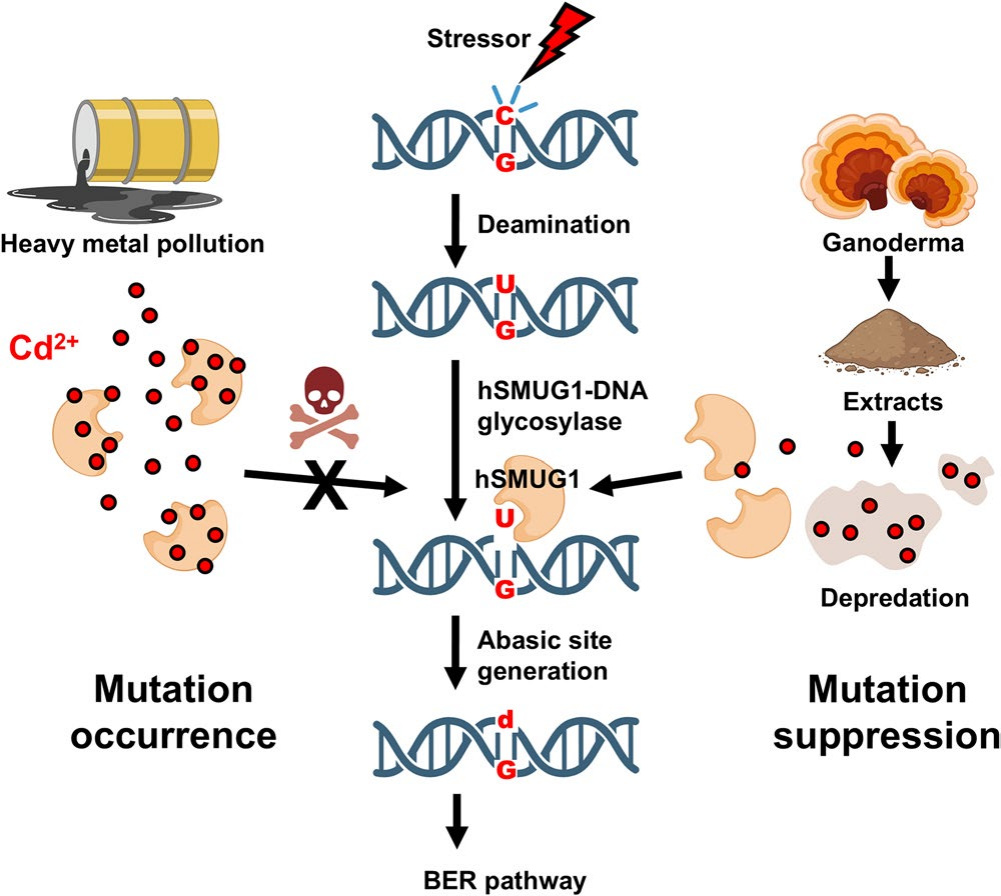
Graphical illustration of the protective role of Ganoderma extract in preserving hSMUG1-mediated uracil excision activity against cadmium-induced inhibition to maintain genomic stability. Upon DNA damage, cytosine bases undergo spontaneous deamination, leading to uracil formation and potential C:G-to-A:T transition mutations. Environmental cadmium ions inhibit hSMUG1 activity, impairing uracil removal, and consequently blocking the activation of the base excision repair (BER) pathway. Treatment with Ganoderma extract effectively attenuates cadmium-induced inhibition, preserves hSMUG1 uracil-excision capacity, and thereby maintains genomic integrity.

In this study, we show that cadmium inhibits hSMUG1-mediated uracil excision in a dose-dependent manner, and that Ganoderma extracts partially restore enzymatic activity under *in vitro* conditions, likely through the reduction of bioavailable cadmium (Figure 7). These findings provide a quantitative framework for assessing the sensitivity of base excision repair enzymes to environmental toxicants. The potential implications for cellular DNA repair or disease-related processes require further investigation using appropriate biological models.

## Data Availability

The datasets generated and/or analysed during the current study are available from the corresponding author upon reasonable request.
